# Onset of the COVID-19 pandemic reduced active time in patients with implanted cardiac devices

**DOI:** 10.1186/s11556-022-00305-0

**Published:** 2022-11-02

**Authors:** Nicholas Sommers, Marcie Berger, Jason C. Rubenstein, James Roth, Amy Pan, Colton Thompson, Michael E. Widlansky

**Affiliations:** 1 Medical College of Wisconsin, Milwaukee, WI USA; 2 Division of Cardiovascular Medicine,, Medical College of Wisconsin, Milwaukee, WI USA

**Keywords:** COVID-19 lockdown, Implantable Devices, Physical inactivity, Sedentary Behavior

## Abstract

**Background:**

Physical inactivity and sedentary behavior are modifiable risk factors for chronic disease and all-cause mortality that may have been negatively impacted by the COVID-19 shutdowns.

**Methods:**

Accelerometry data was retrospectively collected from 332 permanent pacemaker (PPM) and 244 implantable cardiac defibrillation (ICD) patients for 6 time points: March 15-May 15, 2020 (pandemic period), January 1-March 14, 2020, October 1-December 31, 2019, March 15-May 15, 2019, January 1-March 14, 2019, and October 1-December 31, 2018. Paired t-tests, with Bonferroni correction, were used to compare time periods.

**Results:**

Activity significantly decreased during the pandemic period compared to one year prior by an average of 0.53 ± 1.18h/day (P < 0.001) for PPM patients and 0.51 ± 1.2h/day (P < 0.001) for ICD patients. Stratification of subjects by active time (< 2 versus ≥ 2h/day) showed patients with < 2h, particularly those with ICDs, had modestly greater activity reductions with the pandemic onset. Logistical regression analyses suggest a trend toward a greater reduction in active time at the onset of the pandemic and an increased risk of hospital or emergency department (ED) admission for PPM patients, but not ICD patients.

**Conclusion:**

The onset of the pandemic in the United States was associated with a significant drop in PPM and ICD patient active hours that was modestly more pronounced in less active patients and cannot be explained by one year of aging or seasonal variation. If sustained, these populations may experience excess cardiovascular morbidity.

## Background

Over the last decade, numerous studies have demonstrated a dose-dependent relationship between physical activity and a reduction in all-cause mortality, coronary heart disease, and type 2 diabetes [1–7]. Time engaged in sedentary behavior, defined as any waking behavior with an energy expenditure  ≤ 1.5 METs while in a sitting or reclining posture, is a risk factor for cardiovascular disease and mortality, though precise quantitative relationships are not well defined [2, 8−10]. Prolonged sedentary time’s association with increases in inflammatory biomarkers, impaired endothelial function, and insulin resistance may explain these observed adverse cardiovascular outcomes [11, 12]. Therefore, environments and circumstances that drive decreases in physical activity and increases in sedentary behavior may increase cardiometabolic risk. Such circumstances would include the social distancing and stay-at-home orders that began throughout the United States in mid-March 2020.

Readily available accelerometry data from implantable cardiac devices, such as permanent pacemakers (PPM) and implantable cardioverter defibrillators (ICD), offer a unique opportunity to determine the effect of the onset of the COVID-19 pandemic on patient activity. Implanted devices offer objective and repeated measurements of activity in patient populations at high risk for adverse clinical events with regular follow-up prior to the onset and throughout the pandemic. PPMs are implanted in patients with symptomatic irreversible bradycardia or heart block, and ICDs are used for primary and secondary prevention of sudden cardiac death due to ventricular tachycardia. Most patients who receive ICDs have ischemic or non-ischemic cardiomyopathies with left ventricular ejection fractions commonly less than 35% (normal > 50%), whereas most individuals with PPMs have left ventricular ejection factions ≥ 50%. Given divergent clinical indications, there are several significant demographic differences between the two device groups which should therefore be examined independently. Compared with ICD patients, PPM patients are more likely to be female, older, and have significantly fewer comorbidities [13].

Device accelerometer measurements have already been established to predict morbidity and mortality [1, 14, 15]. Tyagi et al. observed that pacemaker patients with < 1h and 1–2h of physical activity per day had a 7.44 and 3.47 times increased risk of death compared to those with > 3h per day [1]. Further, a systematic review of the literature by Rosman et al. 2018 concluded that implanted device-measured physical activity can be useful for predicting clinical outcomes [14]. We leveraged these data supporting the predictive value of device accelerometer measurements to test the hypothesis that the onset of the COVID-19 pandemic would be associated with a significant and sustained decrease in the daily activity of PPM and ICD patients, leading to an increased risk of hospitalization and emergency department (ED) visits.

## Methods

**Subject Selection and Categorization by Active Minutes**: All study procedures were reviewed and approved by the Medical College of Wisconsin’s Institutional Research Board. All subjects with Medtronic (Minneapolis, MN) PPMs and ICDs who underwent a device interrogation during the time period from March 15 to May 15, 2020 and had at least one prior interrogation during one of the following time periods: Jan.1-March 14, 2020; Oct. 1-Dec. 31, 2019; March 15-May 15, 2019; Jan. 1-March 15, 2019; Oct. 1-Dec. 31, 2018, were eligible to be included in these analyses. March 15 to May 15, 2020 was considered the “pandemic period” for the purposes of this study. Clinical data for each eligible subject was extracted from the medical record following a standardized extraction form (Supplemental File 1). Daily active minutes were recorded by device-based accelerometers and reported on device interrogation outputs as an average of the active hours per day for the week prior to device interrogation. For these devices, a minute is classified as active if the individual takes more than approximately 60 steps per minute (estimated to be > 1.5 METS).

### Outcome measurements

We reviewed the electronic medical record (EPIC, Epic Systems, Verona, WI) and recorded whether a subject visited the ED or was hospitalized at our medical center or other local medical centers during the pandemic period. All ED visits and all hospitalizations regardless of the indication were included in this study. We additionally screened the medical record to determine if any subjects were diagnosed with COVID-19 between March 15 and May 15, 2020.

**Statistical Analyses**: Subjects with PPMs and ICDs were analyzed separately given the differences in clinical indications for implantation and population characteristics. PPM and ICD groups were analyzed both without regard to activity level during the early pandemic period (March 15-May 15, 2020) as well as following stratification by whether the individuals averaged < 2h versus ≥ 2h of active time per day during the early pandemic period in the United States (March 15-May 15, 2020). This active time cut-off point was selected based on our prior work showing individuals with PPMs who average < 2h of active time/day are at significantly greater risk of mortality compared to those who averaged ≥ 2h of active time per day[1]. Subject demographic and clinical characteristics between those with < 2 vs. ≥2 active hours/day were compared using unpaired t-tests or Chi-square test as appropriate. We compared active hours between the five time periods prior to the pandemic to the early pandemic period as well as between time periods one year apart using linear mixed effects models. We calculated the differences in active hours between each time period prior to pandemic and early pandemic period. We then performed logistical regression to determine whether changes in active time from any pre-pandemic period to the pandemic period were associated with emergency department visits and hospitalizations. Age at enrollment, total number of comorbidities (creatinine clearance < 60 ml/min, left ventricle ejection fraction < 50%, moderate or more mitral regurgitation, left bundle branch block on ECG, history of coronary artery disease, history of diabetes, history of hypertension, history of dyslipidemia, history of atrial fibrillation, current smoking), and total number of cardiac medications were included as covariates. Unstandardized effect size, test statistics with corresponding degrees of freedom, and standardized effect size (Cohen’s d or partial Eta squared $\left({\eta }_{p}^{2}\right)$) were reported. False discovery rate (FDR) was used to correct for multiple comparisons. P < 0.05 or FDR-adjusted P < 0.05 were considered significant. All statistical analyses were performed using SPSS 24 (IBM Corp., Armonk, NY), SAS 9.4 (SAS Institute Inc., Cary, NC) and R “effectsize” package (please insert citation: Ben-Shachar M, Lüdecke D, Makowski D (2020). effectsize: Estimation of Effect Size Indices and Standardized Parameters. Journal of Open Source Software, 5(56), 2815. doi: 10.21105/joss.02815).

## Results

### Study Population characteristics

**Fig. 1:**
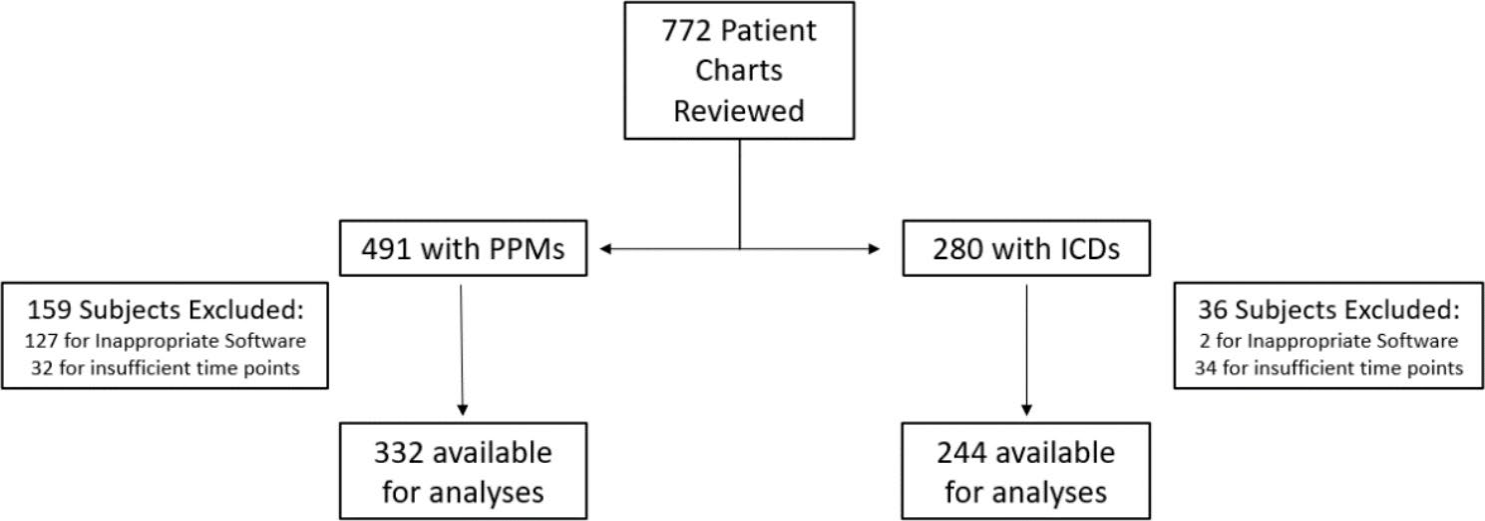
Consort diagram of the study population

We reviewed a total of 491 charts of patients with PPMs and 280 charts of patients with ICDs, respectively. Of these, 332 PPM patients and 244 ICD patients met study inclusion criteria (Fig. 1). Table 1 describes basic demographic information of the PPM patients and Table 2 reports these data for the ICD patient population. When patients with PPMs were stratified by active time (< 2h vs. ≥ 2h), individuals who averaged < 2h/day of active time during the early pandemic period were older (82 ± 10 vs. 73 ± 13 years, N = 128 vs. 204 P < 0.001), more likely to be female (54% vs. 43%, P = 0.04), and more likely to have a creatinine clearance < 60 mL/min (54% vs. 42%, P = 0.04). Those with a history of atrial fibrillation were more likely to have a higher atrial fibrillation burden (16% vs. 9%, P = 0.05).

**Table 1:** Permanent Pacemaker Subject Characteristics

	< 2h active time (N = 128)	≥ 2h active time (N = 204)	P-Value
Age (years)	81.6 ± 9.7	73.4 ± 12.9	< 0.001
Sex (% Women)	54.7	42.6	0.04
Number of Leads			0.75
Single	4.7	3.4	-
Dual	83.6	82.9	-
Biventricular	11.7	13.7	-
Atrial Fibrillation Burden (% of time)	16.0	9.3	0.05
**Co-Morbidities**			
Hypertension (%)	81.3	73.0	0.11
Dyslipidemia (%)	74.2	67.6	0.22
Ischemic Heart Disease (%)	48.4	40.2	0.17
Mitral Regurgitation (% moderate or greater)	12.8	6.7	0.07
History of Atrial Fibrillation (%)	56.3	50.0	0.31
LBBB (%)	16.2	16.4	1.00
LVEF < 35% (%)	3.9	2.5	0.52
Diabetes (%)	29.7	28.4	0.81
Creatinine Clearance < 60 mL/min (%)	53.9	42.2	0.04
Smoking (% current)	5.5	3.9	0.59
**Medications**			
Aspirin (%)	47.7	55.0	0.21
Beta Blockers (%)	69.5	68.3	0.90
Calcium Channel Blockers (%)	26.6	19.8	0.13
ACE Inhibitors (%)	21.1	26.7	0.29
Angiotensin II Receptor Blockers (%)	19.5	25.2	0.43
Angiotensin Receptor/Neprolysin Inhibitor (%)	0.8	1.5	1.00
Nitrates (%)	13.3	14.4	0.87
HMG-CoA Reductase Inhibitor (%)	64.8	64.9	1.00
Amiodarone (%)	7.8	7.9	1.00
Dofetilide (%)	2.4	1.0	0.38
Sotalol (%)	1.6	3.5	0.49
Class I Antiarrhythmics (%)	1.6	2.0	1.00
NOAC (%)	30.5	25.7	0.38
Warfarin (%)	28.1	23.8	0.44

**Table 2:** Implantable Cardioverter Defibrillation Subject Characteristics

	< 2h active time (N = 130)	≥ 2h active time (N = 114)	P-Value
Age	72 ± 12	62 ± 14	< 0.0001
Sex (% Women)	35.4	21.9	0.02
Number of Leads			0.02
Single	23.1	38.6	-
Dual	30.8	28.1	-
Biventricular	46.2	33.3	-
**Comorbidities**			
Hypertension (%)	80.8	66.7	0.01
Dyslipidemia (%)	73.1	62.3	0.07
Ischemic Heart Disease (%)	74.6	56.1	0.002
Mitral Regurgitation (% moderate or greater)	12.0	9.2	0.75
History of Atrial Fibrillation (%)	53.8	45.6	0.20
LBBB (%)	40.0	34.2	0.35
LVEF < 35% (%)	41.5	23.7	0.003
Diabetes (%)	18.9	19.3	0.01
Creatinine Clearance < 60 mL/min (%)	43.8	14.9	< 0.001
Smoking (% current)	14.6	7.9	0.10
**Medications**			
Aspirin (%)	60.0	63.2	0.61
Beta Blockers (%)	94.6	95.6	0.72
Calcium Channel Blockers (%)	10.0	22.8	0.006
ACE Inhibitors (%)	28.5	35.1	0.27
Angiotensin II Receptor Blockers (%)	17.7	21.9	0.41
Angiotensin Receptor/Neprolysin Inhibitor (%)	24.6	14.0	0.04
Nitrates (%)	23.8	15.8	0.12
HMG-CoA Reductase Inhibitor (%)	75.4	63.2	0.04
Amiodarone (%)	19.2	17.5	0.74
Dofetilide (%)	1.5	0.9	1.00
Sotalol (%)	3.1	3.5	1.00
Class I Antiarrhythmics (%)	3.1	5.3	0.52
NOAC (%)	20.0	17.5	0.63
Warfarin(%)	34.6	16.7	0.001

For patients with ICDs, individuals with < 2h of active time per day were more likely to be older (72 ± 12 vs. 62 ± 14 years, N = 130 vs. 114, P < 0.0001), female (35% vs. 22%, P = 0.02), have hypertension (P = 0.01), ischemic heart disease (P = 0.002), LVEF < 35% (P = 0.003), diabetes mellitus (P = 0.01), a creatinine clearance < 60 mL/min (P < 0.001), a biventricular device (P = 0.02), and take warfarin, a HMG-CoA reductase inhibitor, or an ARNI (P = 0.001, 0.04, 0.04, respectively). Individuals with < 2h of active time per day were also less likely to be taking a calcium channel blocker (P = 0.006).

### Impact of the onset of the pandemic in the US on active time in PPM patients

Of the 334 PPM patients included in the study, a total of 63 PPM patients had data for all six time points. The total number of subjects with a data point for each of the 5 non-pandemic time periods were as follows: January-March 2020, N = 296; October-December 2019, N = 250; March-May 2019, N = 183; January-March 2019, N = 180; October-December 2018, N = 186.

In the initial model comparing active time during the pandemic period compared to all other periods including time period, active time < 2h or ≥ 2h, and the interaction of time period and active time < 2h, a significant drop in active time was seen for both individuals who had < 2h of active time [-0.55 ± 0.08h, *t*(1093)=-7.16, Cohen’s d=-0.43, FDR-adjusted P < 0.0001] and those who had ≥ 2h of active time [-0.29 ± 0.06h, *t*(1093)=-4.80, Cohen’s d=-0.29, FDR-adjusted P < 0.0001]. The effect was minimally perturbed when age, number of comorbidities, and number of medications were added into the model as covariates: for those with < 2h of active time [-0.55 ± 0.08h, *t*(1093)=-7.16, Cohen’s d=-0.43, FDR-adjusted P < 0.0001] and those who had ≥ 2h of active time [-0.29 ± 0.06h, *t*(1093)=-4.80, Cohen’s d=-0.29, FDR-adjusted P < 0.0001]. Age [*F*(1, 1093) = 12.99, ${\eta }_{p}^{2}$=0.01, P<0.001] and number of medications (*F*(1, 1093)=4.40, ${\eta }_{p}^{2}$=0.004, P=0.04) were also significantly associated with changes in active time, while number of comorbidities was not associated with changes in active time with the onset of the pandemic [*F*(1, 1093)=2.13, ${\eta }_{p}^{2}$=0.002, P = 0.15].

**Fig. 2:**
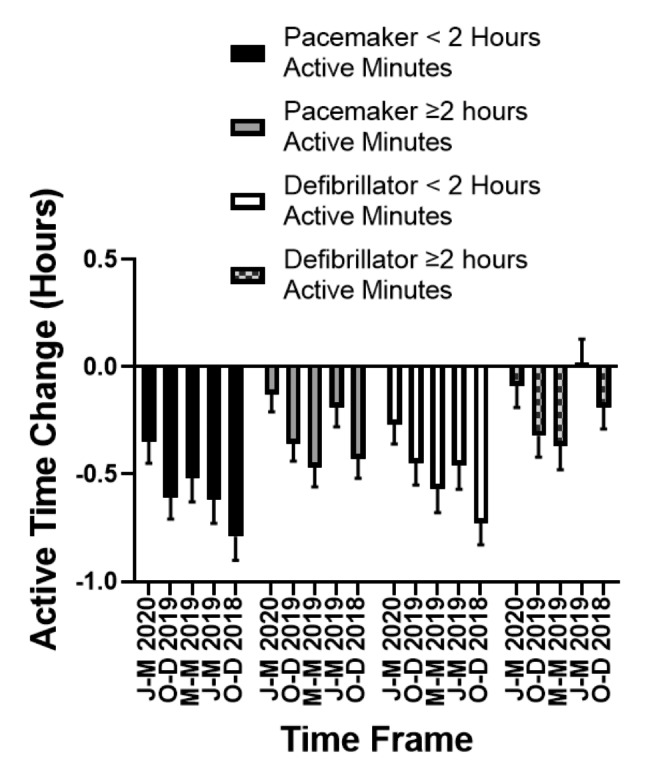
Changes in Active Minutes during the Early Pandemic Period in the United States (March 15-May 15, 2020) in Patients with Pacemakers and Defibrillators. Time differences expressed as the difference in active hours between the early pandemic period and the time frame indicated below each bar. J-M: January-March; O-D: October-December; M-M: March-May.

We constructed additional models to determine whether differences in active time between the early pandemic time period (March 15-May 15, 2020) differed between the five other prior time periods we specifically investigated. In a model that included each time period, active time categorization of < 2h or ≥ 2h, and the interaction of duration and active time category, whether an individual was active ≥ 2h or < 2h (active time category, *F*(1, 1085) = 347.70, ${\eta }_{p}^{2}$=0.24, P<0.0001) and the interaction term for the active minute categories and time period of active time measurement [*F*(1, 1085)=2.62, ${\eta }_{p}^{2}$=0.002, P=0.02] were significantly associated with changes in active time. The changes in active time for each time period compared to the early pandemic period are depicted in the Fig. 2 broken down active time group (< 2h and ≥2h).

Significant drops in active time were seen comparing the early pandemic period to all other time periods measured for those who were active for < 2h on average per day (Table 3). We additionally found there was a significant reduction in active time noted between January 1 and March 15, 2020 versus January 1 and March 15, 2019 for this group (-0.28 ± 0.12h, *t*(1085)=-2.35, Cohen’s d=-0.14, FDR-adjusted P = 0.03). No significant reduction in active time were noted between the other pair of non-pandemic periods that were one year apart [Oct1-Dec 31, 2019 vs. Oct 1-Dec 31, 2018, (-0.18 ± 0.12h, *t*(1085)=-1.56, Cohen’s d=-0.09, FDR-adjusted P = 0.14)]. Following adjustment for age, number of comorbidities, and number of medications, the differences noted remained (Table 4**)**.

**Table 3:** Comparison of Active Time During Early Pandemic Period to Earlier Time Periods in Patients with PPMs.

Time Period Comparison		Estimate mean difference ± SE	DF	t-value	Cohen’s d	FDR-adjusted P value
	**Active Minutes < 2h**					
Mar 15-May 15, 2020 vs. Jan 1-Mar 14, 2020		-0.35 ± 0.10	1085	-3.56	-0.22	< 0.001
Mar 15-May 15, 2020 vs. Oct 1-Dec 31, 2019		-0.61 ± 0.10	1085	-5.94	-0.36	< 0.0001
Mar 15-May 15, 2020 vs. Mar 15-May 15, 2019		-0.52 ± 0.11	1085	-4.54	-0.28	< 0.0001
Mar 15-May 15, 2020 vs. Jan 1-Mar 14, 2019		-0.62 ± 0.11	1085	-5.51	-0.33	< 0.0001
Mar 15-May 15, 2020 vs. Oct 1-Dec 31, 2018		-0.79 ± 0.11	1085	-7.14	-0.43	< 0.0001
Jan 1-Mar 14 2020 vs. Jan 1-Mar 14, 2019		-0.28 ± 0.12	1085	-2.35	-0.14	0.03
Oct 1-Dec 31, 2019 vs. Oct 1-Dec 31, 2018		-0.61 ± 0.12	1085	-1.56	-0.09	0.14
	**Active Minutes ≥ 2h**					
Mar 15-May 15, 2020 vs. Jan 1-Mar 14 2020		-0.13 ± 0.08	1085	-1.69	-0.10	0.12
Mar 15-May 15, 2020 vs. Oct 1-Dec 31, 2019		-0.36 ± 0.08	1085	-4.53	-0.28	< 0.0001
Mar 15-May 15, 2020 vs. Mar 15-May 15, 2019		-0.47 ± 0.09	1085	-5.17	-0.31	< 0.0001
Mar 15-May 15, 2020 vs. Jan 1-Mar 14, 2019		-0.19 ± 0.09	1085	-2.11	-0.13	< 0.05
Mar 15-May 15, 2020 vs. Oct 1-Dec 31, 2018		-0.43 ± 0.09	1085	-4.76	-0.29	< 0.0001
Jan 1-Mar 14, 2020 vs. Jan 1-Mar 14, 2019		-0.07 ± 0.09	1085	-0.70	-0.04	0.48
Oct 1-Dec 31, 2019 vs. Oct 1-Dec 31, 2018		-0.07 ± 0.10	1085	-0.71	-0.04	0.48

**Table 4:** Comparison of Active Time During Early Pandemic Period to Earlier Time Periods in Patients with PPMs with Adjustment for Age, Number of Comorbidities, and Number of Medications

Time Period Comparison		Estimate mean difference ± SE	DF	t-value	Cohen’s d	FDR-adjusted P value
	**Active Minutes < 2h**					
Mar 15-May 15, 2020 vs. Jan 1-Mar 14 2020		-0.35 ± 0.10	1085	-3.57	-0.22	0.0007
Mar 15-May 15, 2020 vs. Oct 1-Dec 31, 2019		-0.61 ± 0.10	1085	-5.92	-0.36	< 0.00001
Mar 15-May 15, 2020 vs. Mar 15-May 15, 2019		-0.52 ± 0.11	1085	-4.54	-0.28	0.00001
Mar 15-May 15, 2020 vs. Jan 1-Mar 14, 2019		-0.62 ± 0.11	1085	-5.49	-0.33	< 0.00001
Mar 15-May 15, 2020 vs. Oct 1-Dec 31, 2018		-0.80 ± 0.11	1085	-7.18	-0.44	< 0.00001
Jan 1-Mar 14 2020 vs. Jan 1-Mar 14, 2019		-0.26 ± 0.12	1085	-2.32	-0.14	0.03
Oct 1-Dec 31, 2019 vs. Oct 1-Dec 31, 2018		-0.19 ± 0.12	1085	-1.61	-0.10	0.13
	**Active Minutes ≥ 2h**					
Mar 15-May 15, 2020 vs. Jan 1-Mar 14 2020		-0.13 ± 0.08	1085	-1.70	-0.10	0.11
Mar 15-May 15, 2020 vs. Oct 1-Dec 31, 2019		-0.37 ± 0.08	1085	-4.57	-0.28	0.00001
Mar 15-May 15, 2020 vs. Mar 15-May 15, 2019		-0.47 ± 0.09	1085	-5.17	-0.31	< 0.00001
Mar 15-May 15, 2020 vs. Jan 1-Mar 14, 2019		-0.19 ± 0.09	1085	-2.06	-0.13	0.06
Mar 15-May 15, 2020 vs. Oct 1-Dec 31, 2018		-0.43 ± 0.09	1085	-4.73	-0.29	0.00001
Jan 1-Mar 14, 2020 vs. Jan 1-Mar 14, 2019		-0.06 ± 0.09	1085	-0.64	-0.04	0.52
Oct 1-Dec 31, 2019 vs. Oct 1-Dec 31, 2018		-0.06 ± 0.10	1085	-0.65	-0.04	0.52

For individuals who performed ≥ 2h of active time, we found no significant difference between the early pandemic time period and the January 1- March 15, 2020 time period (-0.13 ± 0.08h, *t*(1085)=-1.69, Cohen’s d=-0.10, FDR-adjusted P = 0.12, Table 3). Active time for this group was lower during the early pandemic period compared to each of the other four time periods (Table 3**)**. Neither of the paired non-pandemic periods one-year apart showed significant changes in active time for those with active time ≥ 2h (Table 3). Following adjustment for age, number of comorbidities, and number of medications, the significant reduction in active time between the early pandemic period and January 1-March 15, 2019 was no longer significant (-0.19 ± 0.09h, *t*(1085)=-2.06, Cohen’s d=-0.13, FDR-adjusted P = 0.06). All other significant differences between the pandemic period and other time periods remained **(**Table 4).

### Impact of the onset of the pandemic in the US on active time in ICD patients

Of the 244 ICD patients included in the study, a total of 43 ICD patients had data for all six time points. The changes in active time for each time period compared to the early pandemic period are depicted in the Fig. 2 broken down active time group (< 2h and ≥ 2h). The total number of subjects with a data point for each of the 5 non-pandemic time periods were as follows: January-March 2020, N = 186; October-December 2019, N = 169; March-May 2019, N = 132; January-March 2019, N = 129; October-December 2018, N = 159. In the initial model comparing active time during the pandemic period compared to all other periods including time period, active time < 2h or ≥ 2h, and the interaction of time period and active time < 2h, a significant drop in active time was observed for individuals who had < 2h of active time [-0.48 ± 0.07h, *t*(804)=-6.68, Cohen’s d=-0.47, FDR-adjusted P < 0.0001] but not those who had ≥ 2h of active time [-0.14 ± 0.08h, *t*(804)=-1.86, Cohen’s d=-0.13, FDR-adjusted P = 0.06). The interaction was minimally perturbed when age, number of comorbidities, and number of medications were added into the model as covariates for those with < 2h of active time [-0.48 ± 0.07h, *t*(803)=-6.70, Cohen’s d=-0.47, FDR-adjusted P < 0.0001] and those who had ≥ 2h of active time (-0.14 ± 0.08h, *t*(803)=-1.91, Cohen’s d=-0.13, FDR-adjusted P = 0.06)]. Age [*F*(1, 803) = 19.18, ${\eta }_{p}^{2}$=0.02, P<0.0001] was significantly associated with changes in active minute time, while number of comorbidities [*F*(1, 803)=0.49, ${\eta }_{p}^{2}$=0.0006, P=0.49] and number of medications taken [*F*(1, 803)=1.05, ${\eta }_{p}^{2}$=0.001, P = 0.30] were not associated with changes in active time with the onset of the pandemic.

We constructed additional models to determine whether differences in active minutes between the early pandemic time period (March 15-May 15, 2020) differed between the five other prior time periods we specifically investigated. In a model that included each time period, active time categorization of < 2h or ≥ 2h, and the interaction of duration and active time category, whether an individual was active ≥ 2h or < 2h (active time category *F*(1, 796) = 392.70, ${\eta }_{p}^{2}$=0.33, P<0.0001) and the interaction term for the active minute categories and time period of active time measurement (*F*(1, 796)=4.13, ${\eta }_{p}^{2}$=0.005, P=0.001) were significantly associated with changes in active time. Significant drops in active time were seen comparing the early pandemic period to all other time periods measured for those when were active for < 2h on average per day (Table 5). For those who averaged ≥ 2h of active time per day, the early pandemic period active time was significantly lower compared to only the Oct-Dec 2019 (-0.32±0.10h, *t*(796)=-3.15, Cohen’s d=-0.22, FDR-adjusted P = 0.004) and March-May 2019 (-0.37±0.11h, *t*(796)=-3.45, Cohen’s d=-0.24, FDR-adjusted P = 0.002) time periods (Table5).

**Table 5:** Comparison of Active Time During Early Pandemic Period to Earlier Time Periods in Patients with ICDs

Time Period Comparison		Estimate mean difference ± SE	DF	t-value	Cohen’s d	FDR-adjusted P value
	***Active Minutes < 2h***					
Mar 15-May 15, 2020 vs. Jan 1-Mar 14 2020		-0.27 ± 0.092	796	-2.91	-0.21	0.008
Mar 15-May 15, 2020 vs. Oct 1-Dec 31, 2019		-0.45 ± 0.095	796	-4.79	-0.34	0.00001
Mar 15-May 15, 2020 vs. Mar 15-May 15, 2019		-0.57 ± 0.11	796	-5.32	-0.38	< 0.00001
Mar 15-May 15, 2020 vs. Jan 1-Mar 14, 2019		-0.46 ± 0.11	796	-4.30	-0.30	0.00007
Mar 15-May 15, 2020 vs. Oct 1-Dec 31, 2018		-0.73 ± 0.10	796	-7.42	-0.53	< 0.00001
Jan 1-Mar 14 2020 vs. Jan 1-Mar 14, 2019		-0.19 ± 0.11	796	-1.67	-0.12	0.13
Oct 1-Dec 31, 2019 vs. Oct 1-Dec 31, 2018		-0.27 ± 0.11	796	-2.56	-0.18	0.018
	**Active Minutes ≥ 2h**					
Mar 15-May 15, 2020 vs. Jan 1-Mar 14 2020		0.09 ± 0.10	796	0.98	0.07	0.38
Mar 15-May 15, 2020 vs. Oct 1-Dec 31, 2019		-0.32 ± 0.10	796	-3.15	-0.22	0.004
Mar 15-May 15, 2020 vs. Mar 15-May 15, 2019		-0.37 ± 0.11	796	-3.45	-0.24	0.002
Mar 15-May 15, 2020 vs. Jan 1-Mar 14, 2019		0.02 ± 0.11	796	0.22	0.02	0.83
Mar 15-May 15, 2020 vs. Oct 1-Dec 31, 2018		-0.19 ± 0.10	796	-1.90	-0.13	0.09
Jan 1-Mar 14, 2020 vs. Jan 1-Mar 14, 2019		-0.07 ± 0.12	796	-0.61	-0.04	0.59
Oct 1-Dec 31, 2019 vs. Oct 1-Dec 31, 2018		0.12 ± 0.11	796	1.13	0.08	0.33

We also found for those with < 2h active time, there was also significant reduction in active time noted between October 1-December 31, 2019 versus October 1-December 31, 2018 for this group [-0.27 ± 0.11h, *t*(796)=-2.56, Cohen’s d=-0.18, FDR-adjusted P = 0.02]. No significant reduction in active time were noted between the other pair of non-pandemic periods that were one year apart [January 1-March 15, 2020 vs. January 1-March 15, 2019, [-0.19 ± 0.11h, *t*(796)=-1.67, Cohen’s d=-0.12, FDR-adjusted P = 0.13]. Following adjustment for age, number of comorbidities, and number of medications, the differences noted remained for all comparisons (Table 6).

**Table 6:** Comparison of Active Time During Early Pandemic Period to Earlier Time Periods in Patients with ICDs with Adjustment for Age, Number of Comorbidities, and Number of Medications

Time Period Comparison		Estimate mean difference ± SE	DF	t-value	Cohen’s d	FDR-adjusted P value
	***Active Minutes < 2h***					
Mar 15-May 15, 2020 vs. Jan 1-Mar 14 2020		-0.27 ± 0.09	795	-2.92	-0.21	0.007
Mar 15-May 15, 2020 vs. Oct 1-Dec 31, 2019		-0.46 ± 0.09	795	-4.83	-0.34	0.00001
Mar 15-May 15, 2020 vs. Mar 15-May 15, 2019		-0.57 ± 0.11	795	-5.30	-0.38	< 0.00001
Mar 15-May 15, 2020 vs. Jan 1-Mar 14, 2019		-0.46 ± 0.11	795	-4.31	-0.31	0.00006
Mar 15-May 15, 2020 vs. Oct 1-Dec 31, 2018		-0.73 ± 0.10	795	-7.43	-0.53	< 0.00001
Jan 1-Mar 14 2020 vs. Jan 1-Mar 14, 2019		-0.19 ± 0.11	795	-1.67	-0.12	0.13
Oct 1-Dec 31, 2019 vs. Oct 1-Dec 31, 2018		-0.27 ± 0.11	795	-2.53	-0.18	0.02
	**Active Minutes ≥ 2h**					
Mar 15-May 15, 2020 vs. Jan 1-Mar 14 2020		0.09 ± 0.10	795	0.93	0.07	0.41
Mar 15-May 15, 2020 vs. Oct 1-Dec 31, 2019		-0.32 ± 0.10	795	-3.18	-0.23	0.004
Mar 15-May 15, 2020 vs. Mar 15-May 15, 2019		-0.37 ± 0.11	795	-3.50	-0.25	0.001
Mar 15-May 15, 2020 vs. Jan 1-Mar 14, 2019		0.02 ± 0.11	795	0.18	0.01	0.85
Mar 15-May 15, 2020 vs. Oct 1-Dec 31, 2018		-0.20 ± 0.10	795	-1.93	-0.14	0.08
Jan 1-Mar 14, 2020 vs. Jan 1-Mar 14, 2019		-0.07 ± 0.12	795	-0.60	-0.04	0.59
Oct 1-Dec 31, 2019 vs. Oct 1-Dec 31, 2018		0.12 ± 0.11	795	1.13	0.08	0.33

For individuals who performed ≥ 2h of active, neither of the paired non-pandemic periods one-year apart showed significant changes in active time for those with active time ≥ 2h (Table 5). Following adjustment for age, number of comorbidities, and number of medications, no significant changes were seen in comparison to the previous model (Table 6).

### Impact of the onset of the pandemic in the US on admissions and ED visits for PPM and ICD patients

During the first two months of the pandemic period, 51 PPM (15.3%) and 37 ICD (13.1%) patients were admitted to the hospital or ED. Patients with < 2h of activity per day were more frequently admitted to the hospital or ED (PPM 19.7% and ICD 19.2%) than those with ≥ 2h (PPM 12.7% and ICD 10.5%), though these differences were not statistically significant (P = 0.06). Notably, during the measured pandemic period, 2 PPM and 0 ICD patients tested positive for COVID.

For PPM patients, in univariate models including only changes in active minutes as an independent variable, changes in active minutes compared to the early pandemic period were only associated with an increased risk of an ED visit or hospitalization when compared to the Oct 1-Dec 31, 2019 and Jan 1-March 14, 2019 time periods (Table 7). These associations remained following adjustment for age, number of comorbidities, and number of medications (Table 7). The interaction terms for active time category (< 2h or ≥ 2h) with change in active minutes was not significant for any comparison (data not shown).

**Table 7:** Logistical Regression Models to Determine Whether Changes in Active Minutes Between the Early Pandemic Period and Five Earlier Time Periods are Associated with an Increased Risk of Hospitalization or an Emergency Department Visit for Patients with PPMs

Time Period	OR	95% CI Lower Limit	95% CI Upper Limit
Univariate Model: Difference in Active Minutes Only			
Jan 1-Mar 14 2020	1.22	0.84	1.77
Oct 1-Dec 31, 2019	***1.43***	***1.05***	***1.95***
Mar 15-May 15, 2019	1.27	0.90	1.78
Jan 1-Mar 14, 2019	***1.70***	***1.12***	***2.59***
Oct 1-Dec 31, 2018	1.36	0.94	1.97
Multivariable Model: Difference in Active Minutes with additional adjustment for age, number of comorbidities, and number of medications taken			
Jan 1-Mar 14 2020	1.15	0.77	1.73
Oct 1-Dec 31, 2019	***1.49***	***1.09***	***2.06***
Mar 15-May 15, 2019	1.30	0.91	1.86
Jan 1-Mar 14, 2019	***1.62***	***1.04***	***2.53***
Oct 1-Dec 31, 2018	1.34	0.91	1.96

For ICD patients, in models that include the change in active minutes, < 2h or ≥ 2h of active time, and an interaction term, the change in active minutes was not predictive of an emergency room visit or hospitalization for any comparison (Table 8).

**Table 8:** Logistical Regression Models to Determine Whether Changes in Active Minutes Between the Early Pandemic Period and Five Earlier Time Periods are Associated with an Increased Risk of Hospitalization or an Emergency Department Visit for Patients with ICDs

Time Period	OR	95% CI Lower Limit	95% CI Upper Limit
Univariate Model: Difference in Active Minutes Only			
Jan 1-Mar 14 2020	0.99	0.67	1.46
Oct 1-Dec 31, 2019	1.13	0.69	1.85
Mar 15-May 15, 2019	1.20	0.82	1.76
Jan 1-Mar 14, 2019	1.06	0.63	1.76
Oct 1-Dec 31, 2018	1.34	0.92	1.93
Multivariable Model: Difference in Active Minutes with additional adjustment for age, number of comorbidities, and number of medications taken			
Jan 1-Mar 14 2020	1.01	0.68	1.52
Oct 1-Dec 31, 2019	1.18	0.71	1.99
Mar 15-May 15, 2019	1.21	0.82	1.79
Jan 1-Mar 14, 2019	1.09	0.64	1.85
Oct 1-Dec 31, 2018	1.32	0.91	1.92

## Discussion

In this study, we found that for patients with PPMs and ICDs, the onset of the pandemic in the United States was associated with a significant reduction of active time, which appeared modestly more accentuated in individuals who averaged < 2h of active time. On average, subjects lost about 3.7h per week of activity of at least 1.5 METs and replaced this activity with more sedentary behavior, potentially increasing risks to their health [11, 12]. The observed reduction of active time cannot be explained by a year of aging or by seasonal variation. While we did not find a consistent increase in the risk of emergency department visits and hospitalizations with reductions on active minutes during the early pandemic period compared to all of the previous time periods investigated, the magnitude of the drop in active time in patients with PPMs, but not with ICDs, tended to be associated with greater risk of these adverse outcomes. These findings with respect to hospital and emergency department admissions were not driven by COVID diagnoses. Our study is the first, to our knowledge, to examine an association between reduced physical activity at the onset of the pandemic and hospital and ED admissions. Together with prior work showing reduced activity levels are associated with higher risk in populations with PPMs and ICDs [1, 15−17], our data suggest the pandemic may lead to increased burden of adverse events and mortality events in the future, particularly if the losses in activity are not regained.

Data collected globally from smart phones demonstrated variable drops in activity levels with the onset of the pandemic [18]. Dozens of publications have reported reductions in activity levels with the onset of the pandemic using self-reported data in young children and college students [19–30] as well as adult populations [31–55]. These data are limited by virtue of being self-reported rather than objectively measured. To our knowledge, only a handful of studies have shown objective evidence of decreases in activity at the onset of the pandemic. One study used self-reported step-count data from each subject’s own, non-standardized smart device-based accelerometer [36]. Four Italian studies (individual sample sizes of 24, 180, 184, and 349 subjects) examining pandemic ICD accelerometry data observed reduced activity levels independent of pre-pandemic activity [56–59]. Our implantable cardiac device patients demonstrated more sedentary time as a result of the pandemic, mirroring the Italian experience, though they were subject to less strict lockdown mandates in the United States. Two additional studies have recently been published with larger data sets containing both ICD and PPM data, one from North Carolina (N = 3453) and another from New York and Minnesota (N = 9924) [60, 61]. Both studies observed a decline in physical activity during the pandemic lockdowns, with activity remaining low several months after restrictions were lifted [60, 61]. Our data adds to this growing body of literature, providing further objective evidence documenting the negative impact of the pandemic on individual activity levels. Our data extend these reports with data suggesting that, in both ICD and PPM cohorts, lower baseline activity level is associated with a modestly greater decline in activity with the onset of the pandemic. This finding contrasts with the North Carolina study that found those who were more active prior to the lockdown were more likely to have physical activity declines [60]. Moreover, Malanchini et al. reported that pre-pandemic activity in Italy did not predict reduction in pandemic activity levels. The discrepancy between our three studies is interesting and the reasons for this are not clear. The disparity may be related to differences in study population composition and geolocation. Particularly for older adults, and the frail tendencies of the cardiac device population, the more challenging climate for outdoor activities in late winter and early spring in Wisconsin may account for part of the greater limitation in activity in patients in our data set.

Our data identified that the onset of the pandemic led to a trend toward increased hospitalizations and emergency department visits in PPM patients associated with greater reductions in active time but not those with ICDs. To our knowledge, no studies to date have found an association between the decline in physical activity at the onset of the pandemic shutdowns and increased hospitalizations or death [60]. Our PPM data generally agrees with prior research that shows decreased physical activity is associated with increased rates of hospitalization [62]. It is not clear why a similar trend was not observed in our ICD subjects. Given underlying substantial structural heart disease in the ICD cohort, predisposing to arrhythmias and heart failure hospitalizations, our sample size and length of follow-up may have been insufficient to establish a relationship between the drop in active time and adverse outcomes. This finding merits additional investigation. Persistent functional decline has been shown in a significant number of older adults patients after 1 year of increased inactivity such as after a major, nonemergent abdominal or thoracic surgery as well as in the months following the COVID lockdowns [60, 61, 63]. Whether more robust evidence will be found linking reduced activity levels due to COVID-19 lockdowns and significant adverse events remains to be seen and warrants additional consideration.

Our PPM and ICD study population demographics, both overall and when stratified by active time, follow observed trends in the literature that have described associations between decreased physical activity and female gender, increasing age, and several comorbidities [2–4, 64−72]. The consistency of our demographic findings with prior activity research increases our confidence in the ability to generalize the data to other similar populations.

Our study has several limitations. First, our data only suggest associations between drops in activity levels and adverse events and should not be construed as a causal relationship. Future studies that focus on interventions to increase physical activity in these populations would help determine if reversing the reduction in active time has a causal and favorable effect on health. Second, patient patterns of seeking care during the pandemic may have been altered due to a fear of exposure to COVID at medical centers. Third, only a minority of subjects (63 with PPMs, 43 with ICDs) had device interrogations during all six time periods evaluated in the study. Repeated measures ANOVA of these data confirm our reported findings but had limited power due to low numbers (data not shown). Counterbalancing these weaknesses are the objective nature of the capture of the activity data, the novel report of baseline sedentary individuals being more heavily affected, and our original data showing an association between reduced activity levels and early adverse events in this population.

## Conclusion

In conclusion, our study shows the onset of the early COVID-19 pandemic shutdowns in the United States in March 2020 was associated with a significant drop in PPM and ICD patient active hours, which was modestly more pronounced in less active patients and cannot be explained by a year of aging or seasonal variation. Additionally, reduced active hours with the onset of the pandemic was associated with a trend towards an increase in the odds of hospital or ED admission in PPM patients. Given what is known about the detrimental effects of reduced active time and elevated sedentary time on the health and well-being of older adults, these data could be a harbinger of increased adverse events in those individuals whose activity levels significantly dropped early in the pandemic. Efforts to increase activity levels in these patients may be warranted to mitigate risk. Future studies evaluating patients’ activity levels and health outcomes post-pandemic will provide additional insight into the long-term consequences of the COVID-19 shutdown.

## Data Availability

The datasets used and/or analyzed during the current study are available from the corresponding author on reasonable request.

## List of abbreviations

PPM: Permanent pacemaker
ICD: Implantable cardioverter defibrillator
ED: Emergency Department
